# Surgeon-Stratified Periprosthetic Fracture Risk in a Single-Hospital Cohort of 1531 Uncemented ABG-II Femoral Stems at Primary Total Hip Arthroplasty

**DOI:** 10.1007/s43465-023-00996-2

**Published:** 2023-09-19

**Authors:** Luka Kropivšek, Vane Antolič, Blaž Mavčič

**Affiliations:** 1 Chair of Orthopaedics, Faculty of Medicine, University of Ljubljana, Zaloška 9, 1000 Ljubljana, Slovenia; 2 Department of Orthopaedic Surgery, University Medical Centre Ljubljana, Zaloška 9, 1000 Ljubljana, Slovenia

**Keywords:** Total hip arthroplasty, Periprosthetic fracture, Cohort analysis, Surgeon stratification

## Abstract

**Purpose:**

Late periprosthetic fracture risk with uncemented ABG-II femoral stems at primary total hip arthroplasty (THA) has been reported before, but single-hospital surgeon-stratified reports of this implant have never been published. We asked whether periprosthetic fracture rates of ABG-II femoral stems implanted at a single tertiary hospital depended on patients’ age, gender and the operating surgeon.

**Methods:**

The study included 1531 consecutive primary ABG-II femoral stems implanted at a single tertiary hospital between January 1, 2012 and December 31, 2018. The Kaplan–Meier and Cox regression analyses were performed after 3.6–10.6 years of follow-up.

**Results:**

In the cohort, we recorded 8 intraoperative, 22 early postoperative (within 90 days of implantation) and 26 late periprosthetic fractures (over 90 days postoperatively). The revision rate of ABG-II femoral stems was 5.1/100 component-years for early and 0.3/100 component-years for late periprosthetic fractures. The Kaplan–Meier cumulative probability of periprosthetic fracture was 2.1% at one, 2.3% at 2, 3.2% at 5, and 6.5% at 10 years after the implantation. Higher patient's age at operation was an independent risk factor of subsequent periprosthetic fracture (hazard ratio 1.07, 95% confidence interval 1.03–1.10; *p* < 0.01), regardless of the operating surgeon. Most of the fractured femora were Dorr type C (stovepipe).

**Conclusion:**

The study presents the largest published ABG-II femoral stem cohort from a single hospital so far with 9291 component-years of observation. Periprosthetic fracture risk of ABG-II increased with patients’ age, had no variability between different surgeons, and was considerably higher from other uncemented femoral stems used at the same hospital.

**Level of Evidence:**

III.

## Introduction

Uncemented hip arthroplasties emerged in the 1970s as an alternative to cemented endoprostheses. In the late 1980s, the designs were improved with the introduction of coated surfaces, new alloys, hemispheric acetabular cups, etc. [1]. The uncemented ABG-II (Anatomic Benoist Girard II) consists of femoral and acetabular component (Stryker Orthopaedics, Mahwah, NJ, USA). The femoral stem is made of titanium TMZF alloy (Titanium, Molybdenum, Zirconium and Ferrous) with the caput–collum–diaphyseal angle of 130°, the taper standardized to V40, short small-diameter polished stem, porous plasma spray coating and hydroxyapatite surface preparation to induce bone apposition and ingrowth. The anatomical stem has built in 12° of anteversion [2, 3]. It is combined with the hemispheric acetabular component, made of titanium alloy (TiAl_6_V_4_) and coated with hydroxyapatite [4]. The ABG-II hip endoprosthesis was introduced in 1996, as the successor of the ABG-I prosthesis, and is frequently used in Europe. The ABG-II femoral component retained the initial ABG-I design, but the new implant was made of a low modulus titanium alloy with shorter small-diameter polished stem, increased proximal hydroxyapatite coating and neck lateralization [1, 4].

Clinical results of ABG-II hip endoprosthesis have been reported extensively. The studies encompassed either arthroplasty registry reports with pooled data from different hospitals [2, 3, 5], multiple hospitals [6] or a single hospital [1, 3, 4, 7–10]. The largest published ABG-II clinical series from a single hospital so far included 587 cases with 12-year review period and pointed out high risk of late periprosthetic fractures with this implant [3]. However, all these reports ignored a very important confounding variable—the surgeon. Surgeon-stratified studies about individual implants are rare. No study to date has analyzed the impact of different orthopaedic surgeons on the long-term clinical outcome of more than thousand ABG-II femoral stems implanted at a single hospital.

The aim of this single-hospital cohort analysis of 1531 consecutive patients with implanted ABG-II femoral stems was to determine whether periprosthetic fracture rates of this implant depended on patients’ age, gender and operating surgeon at primary implantation.

## Patients and Methods

### Patients

The retrospective observational cohort of patients included all implanted primary total hip arthroplasties (1531 hips) with uncemented ABG-II femoral stem (combined with either acetabular cup ABG-II or acetabulum from another manufacturer) between January 1, 2012, and December 31, 2018, at a single tertiary hospital (University Medical Centre Ljubljana, Department of Orthopaedic Surgery, Ljubljana, Slovenia). None of the patients with the primary THA were excluded from the study. Patient data were collected prospectively and were analyzed up to 10 years after the start of the observation period.

### Surgical Technique

Patients were operated under spinal or general anesthesia, in supine position with the direct lateral approach, or in the lateral decubitus position with posterior approach to the hip joint. All surgical procedures were performed in one of the two operating rooms of the same operating suite of a single tertiary university hospital. Perioperative antibiotic prophylaxis, thromboembolic prophylaxis and postoperative rehabilitation protocol were uniform for all patients at a given time-point, but they have been changing between 2012 and 2018 in accordance with the national guidelines. The study cohort patients were followed up from the initial primary total hip arthroplasty until eventual outcome assessment on July 31, 2022.

### Data Acquisition

All cases of revision operations, removals of femoral stem or death were recorded and identified for each of the patients from the hospital records. The patient data were extracted from the surgery protocol archive and included patient’s name and surname, date of birth, gender, date of primary THA, implanted components, side of the operated hip (right/left), operating surgeon and surgical approach at primary THA, dates of all revision operations with indications for revision, identified deep infection at any time-point after the primary THA (yes/no), femoral periprosthetic fracture at any postoperative time-point (yes/no), removal of femoral stem at any postoperative time-point (yes/no), and general health status on July 31, 2022 (alive, deceased with the date of death, missing with the date of last known follow-up). For each of the identified cases with a periprosthetic fracture of the proximal femur, preoperative anterior–posterior radiograph was evaluated for proximal femoral morphology according to Dorr et al. [11] and the postoperative anterior–posterior radiograph was measured for the proximal femoral fit ratio according to Kim et al. [9, 12].

### Statistical Analysis

Statistical analysis was performed with IBM SPSS Statistics 23.0 for Windows (IBM Corp. Armonk, New York, USA). The follow-up period started on the day of implantation of the primary total hip endoprosthesis and ended on the day of endoprosthesis revision for any reason, removal of femoral stem, death, or the last available follow-up date of July 31, 2022. Fisher’s exact test was used to compute the difference in overall revision rates between patients operated with the direct lateral approach and the posterior approach. The Kaplan–Meier method was used to calculate the cumulative proportion of surviving ABG-II femoral stems until the first revision or removal at 5/10 years after the primary THA. With the Cox regression analysis, we assessed the impact of covariables on the ABG-II femoral stem survival until the first revision for any reason, femoral stem removal or periprosthetic fracture as the endpoint. The input covariables included: age of the patient at the time of the primary THA as a numerical variable, gender and the surgeon (consecutively labeled 0–7, comprising of 7 individual experienced surgeons and the reference group 0 representing pooled patients from the 10 less-experienced surgeons who performed less than 50 primary ABG-II femoral stems each). In accordance with the institutional regulations, all surgeries performed by less-experienced surgeons were directly supervised by senior consultants who assisted the surgical procedures.

## Results

The mean age of the cohort of 1350 patients (181 cases were bilateral) was 67.1 ± 10.0 years (range 23.4−89.2 years). There were 765 endoprostheses implanted in men and 766 in women, 865 right and 666 left hips. In 134 cases, only the femoral stem ABG-II was implanted, and in the remaining 1397 cases, both components of ABG-II hip endoprosthesis were implanted.

Altogether, the study included 9291 component-years of observation and the mean follow-up of patients with unrevised ABG-II femoral stems who were alive at the end of the observation period was 6.7 ± 1.9 years (range 3.6–10.6 years) after the primary implantation. At the end the observation period, 1265 (82.6%) cases were confirmed alive with unrevised implant and 146 (9.5%) cases were confirmed dead with unrevised implant. The remaining 120 (7.8%) ABG-II femoral stems had at least one subsequent surgical revision on average 1.9 years after the primary total hip arthroplasty; therefrom, 85 cases had the femoral stem eventually removed and 35 femoral stems were retained. The total number of femoral stems requiring revision for any reason was therefore 1.29 per 100 observed component-years.

**Fig. 1:**
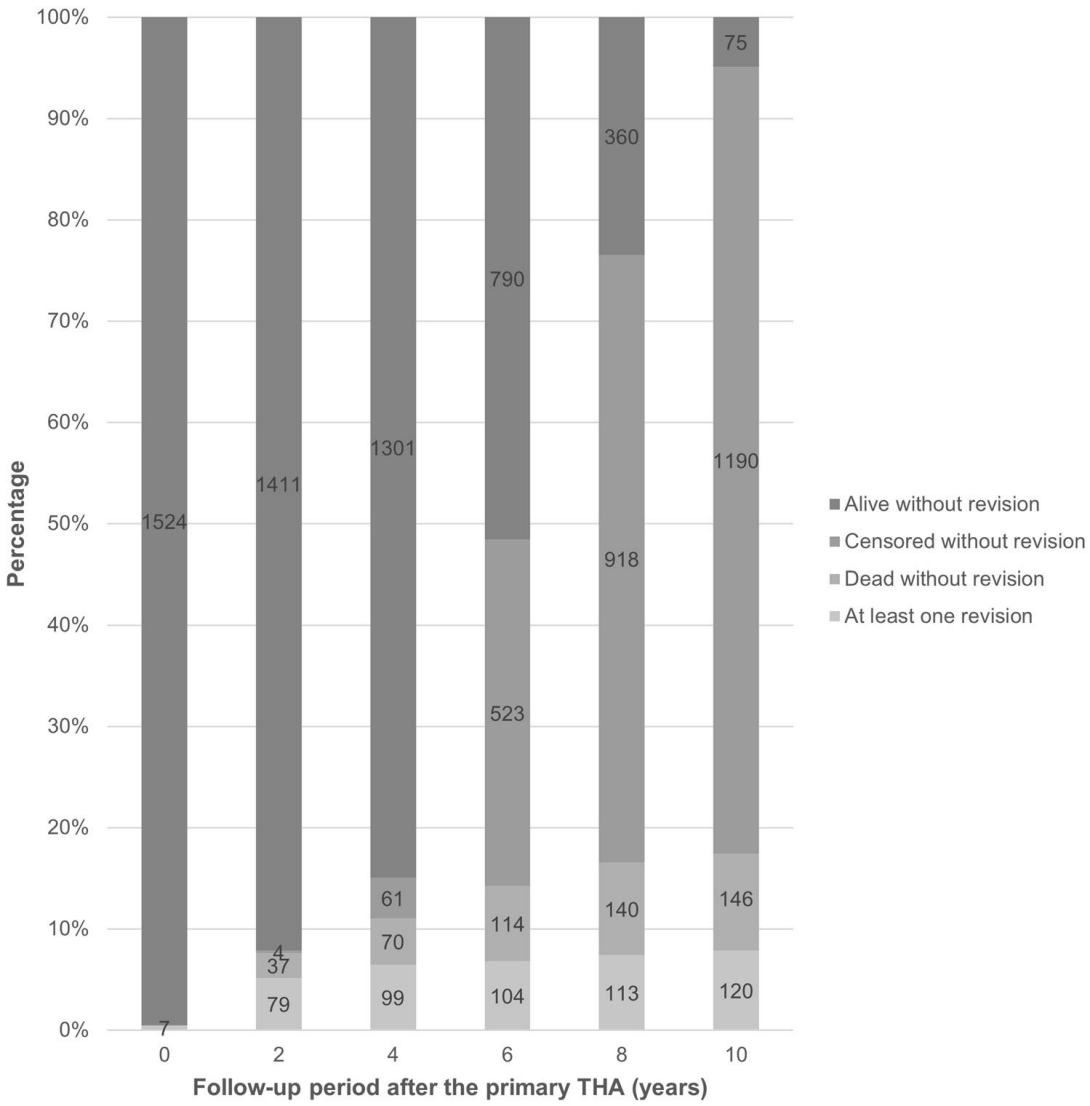
Distribution timeline with numbers of the ABG-II femoral stems cohort subjects who were confirmed alive without revision, censored without revision, confirmed dead without revision, or required at least one revision at 0/2/4/6/8/10 years after the primary THA (total *N* = 1531)

The most common cause for revision operation was periprosthetic fracture, followed by verified bacterial infection (Table 1). In addition to these, 8 periprosthetic fractures (0.5% of the entire cohort) occurred intraoperatively and were treated with cerclage without the need for additional surgical procedure. There was no statistically significant difference in overall revision rates between patients operated with the direct lateral approach or the posterior approach (70 out of 780 vs. 50 out of 751; *p* = 0.106). Early postoperative periprosthetic femoral fractures (i.e., intraoperative or within 90 days of the primary implantation) were recorded in 30 patients (2.0% of the entire cohort) at the mean age of 70.8 ± 7.6 years (range 48.9−84.3 years), while late postoperative periprosthetic femoral fractures over 90 days after the primary implantation were identified in additional 26 patients (1.7% of the entire cohort) who were on average 73.3 ± 6.3 years (range 61.9−84.8 years) old at the time of primary THA and 78.1 ± 6.4 years (range 67.8−90.6 years) old when the periprosthetic fracture occurred (see Fig. 1).

**Table 1:** Clinical results of the 1531 ABG-II femoral stems cohort, implanted at a single tertiary hospital between January 1, 2012 and December 31, 2018 and followed up until July 31, 2022

ABG-II femoral stems		Number of cases	Percentage
Number of the all implanted femoral stems		1531	
Number (percentage) of living patients without revision		1265	(82.6%)
Number (percentage) of deceased patients without revision		146	(9.5%)
Number (percentage) of cases with at least one revision		120	(7.8%)
1	Aseptic loosening of acetabular component only	3	
2	Aseptic loosening of femoral component only	11	
3	Aseptic loosening of both components	1	
4	Verified bacterial infection	32	
5	Hematoma without infection	11	
6	Periprosthetic fracture	48	
7	Wear of the components or mechanical complication	0	
8	Revision of a non-ABG-II component	0	
9	Dislocation	10	
10	Other or unknown	5

**Fig. 2:**
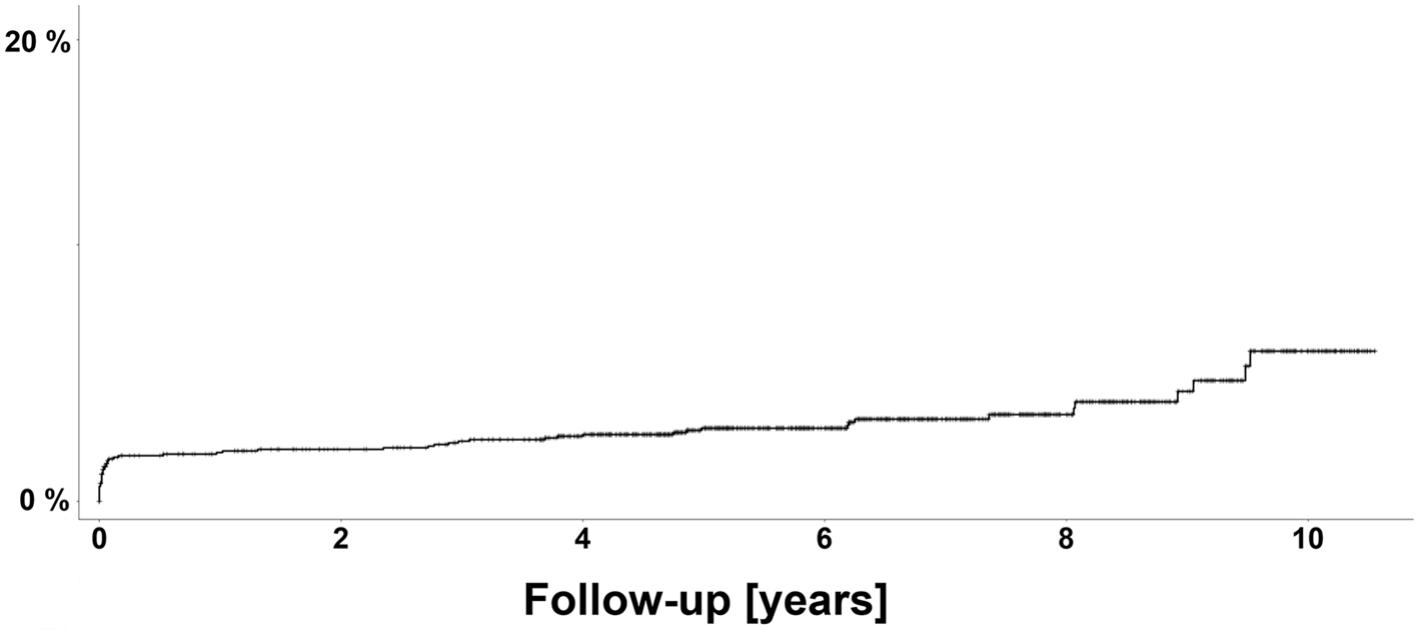
The Kaplan–Meier cumulative probability of periprosthetic fracture in the cohort of 1531 consecutive primary uncemented ABG-II femoral stems

The revision rate of ABG-II femoral stems due to non-intraoperative early periprosthetic fracture (i.e., within 90 days of the primary implantation) was 5.1/100 component-years and the revision rate of late periprosthetic femoral fractures over 90 days postoperatively was 0.3/100 component-years. The Kaplan–Meier cumulative probability of periprosthetic fracture of implanted ABG-II femoral stem was 2.1% at 1 year, 2.3% at 2 years, 3.2% at 5 years and 6.5% at 10 years after the implantation (Fig. 2). Out of the 56 identified cases with a periprosthetic fracture of the proximal femur, 6 were classified on preoperative radiographs as a champagne-flute femur configuration (Dorr type A), 19 had normal femoral shape (Dorr type B), and the remaining 31 were classified as a stovepipe femur (Dorr type C) [11]. The mean proximal femoral fill ratio on the postoperative radiographs of the 56 hips who had a periprosthetic fracture was 0.75, whereby 34 of them hat the femoral fill ratio < 0.80, i.e., classified as non-tight [9, 12]. Only 5 patients were involved in a high-impact traffic accident, the remaining majority suffered a fall from the standing height.

After adjustment for age and gender of the patient, there was no statistically significant difference between orthopaedic surgeons in survival of ABG-II femoral stems until the first revision, survival until implant removal or survival until the first periprosthetic fracture. However, higher patient's age at operation was an independent predictor of subsequent periprosthetic fracture at any time-point (Table 2) with hazard ratio 1.07 for each additional year of age (95% confidence interval 1.03–1.10; *p* < 0.01) and a statistically significant risk factor of late periprosthetic fracture over 90 days after the implantation (Table 3) with hazard ratio 1.10 for each additional year of age (95% confidence interval 1.05–1.17; *p* < 0.01).

**Table 2:** Cox regression analysis of survival until any periprosthetic fracture for the entire cohort of 1531 ABG-II femoral stems, omnibus test of model coefficients *p* = 0.028

	*B*	SE	Exp(B)	95.0% CI for Exp(B)		*p* value
				Lower	Upper	
Age [per year]	0.064	0.017	1.066	1.031	1.103	0.000*
Gender [male = 1]	0.376	0.277	1.456	0.845	2.507	0.176
Surgeon						0.912
Surgeon (1)	– 0.383	0.630	0.682	0.198	2.343	0.543
Surgeon (2)	– 0.162	0.654	0.850	0.236	3.062	0.804
Surgeon (3)	0.040	0.647	1.040	0.293	3.699	0.951
Surgeon (4)	– 0.295	0.692	0.744	0.192	2.891	0.670
Surgeon (5)	– 0.327	0.817	0.721	0.145	3.579	0.689
Surgeon (6)	– 1.279	1.157	0.278	0.029	2.690	0.269
Surgeon (7)	– 0.411	0.915	0.663	0.110	3.986	0.653

Statistically significant *p* values were marked with an asterisk (*)

*B* Cox coefficient, *SE* standard error, *Exp(B)* risk for a revision, *CI* confidence interval, *F+A* femoral and acetabular

**Table 3:** Cox regression analysis of survival until the late periprosthetic fracture (i.e., over 90 days after the primary implantation) for the entire cohort of 1531 ABG-II femoral stems, omnibus test of model coefficients *p* = 0.017

	*B*	SE	Exp(B)	95.0% CI for Exp(B)		*P* value
				Lower	Upper	
Age [per year]	0.100	0.028	1.105	1.045	1.169	0.000*
Gender [male = 1]	0.118	0.399	1.125	0.514	2.460	0.768
Surgeon						0.438
Surgeon (1)	– 1.397	0.845	0.247	0.047	1.296	0.098
Surgeon (2)	– 0.637	0.843	0.529	0.101	2.762	0.450
Surgeon (3)	– 0.773	0.872	0.461	0.084	2.549	0.375
Surgeon (4)	– 0.031	0.824	0.969	0.193	4.871	0.970
Surgeon (5)	0.021	0.915	1.021	0.170	6.140	0.982
Surgeon (6)	– 12.968	367.174	0.000	0.000	0.000	0.972
Surgeon (7)	– 0.749	1.232	0.473	0.042	5.287	0.543

Statistically significant p values were marked with an asterisk (*)

*B* Cox coefficient, *SE* standard error, *Exp(B)* risk for a revision, *CI* confidence interval, *F+A* femoral and acetabular

The periprosthetic fracture rate of ABG-II femoral stems was further compared to the previously published results of uncemented femoral stems Zweymüller SL-PLUS and EcoFit, used in the same tertiary hospital by the same group of surgeons [13, 14]. It turned out the ABG-II femoral stem periprosthetic fracture rate 0.52 per 100 observed component-years (i.e., 48 postoperative periprosthetic fractures after 9291 component-years) in the presented study was considerably higher than Zweymüller SL-PLUS with 0.05 per 100 observed component-years (i.e., 11 postoperative periprosthetic fractures after 23,255 component-years [13]) or EcoFit with 0.19 per 100 observed component-years (i.e., 11 postoperative periprosthetic fractures after 5851 component-years [14]) and the difference was statistically significant *p* < 0.01 for both comparisons.

## Discussion

The ABG-II hip endoprosthesis has been used for a few decades and its clinical results are documented in several studies. However, to the best of the authors’ knowledge, no report to date has analyzed the impact of different orthopaedic surgeons on the periprosthetic fracture risk of more than thousand ABG-II cases operated at a single hospital.

The mean age of patients with inserted ABG-II hip endoprosthesis is comparable with other uncemented hip endoprostheses in the age range of 50–70 years [1, 2, 4, 6, 9, 13]. Most previous reports assessed survival rates of ABG-II endoprosthesis until the first revision with rates in the 94–100% range at approximately 6 years [2, 10], 94–98% range at around 10 years [1, 4], and up to 96.1% at 14 years [6] of follow-up. Results of the presented study showed the survival rates of 93% at 5 years and 89% at 10 years without any revision operation, which is slightly lower compared to the previously published studies mentioned above. Therefore, the survival rate of the entire cohort of ABG-II femoral stems implanted at the Department of Orthopaedic Surgery, University Medical Centre Ljubljana in the first 5–10 years after insertion is comparable to the survival rate of this hip endoprosthesis in the previously published orthopaedic literature and our hypothesis was confirmed. Furthermore, the results of ABG-II femoral stem in the presented cohort of this study were in accordance with the previous findings of arthroplasty registries on the ABG-II hip system and just at the upper limit of still acceptable threshold 1.29 revisions per 100 component-years in the literature [15].

Revision rates due to periprosthetic fractures in the presented ABG-II femoral stem cohort were considerably higher from other uncemented femoral stems used in the same hospital by the same group of surgeons (University Medical Centre Ljubljana, Department of Orthopaedic Surgery) [13, 14] in arthroplasty registries worldwide [16, 17] or in other implant types [18–20]. The design setting of our study enabled adjustment for variability between surgeons in the multivariate analysis and clearly showed that the high ABG-II periprosthetic fracture rate was neither surgeon-dependent nor hospital-specific. In addition, it should not be overlooked that almost half of the postoperative periprosthetic fractures in the presented series occurred far more than 90 days after the primary THA (on average 5 years later) and therefore cannot be directly related to possible intraoperative mishandling of implant. In previously published reports on ABG-II hip endoprosthesis, the most frequent causes of revision operations included fracture followed by loosening [3], loosening [10], dislocation and fracture [6], postoperative infection [1] and periprosthetic fracture [2]. Some reports have determined an increased risk of periprosthetic fractures of ABG-II femoral stem, but our study is the first to exclude the influence of different surgeons and to analyze all implants from the same tertiary hospital [5, 21, 22]. The latest report from the Australian Orthopaedic Association National Joint Replacement Registry and a local hospital also states that the most common late failure of ABG-II implant is periprosthetic fracture [3]. However, our study encompasses a significantly higher number of cases from the same orthopaedic hospital and our results confirm the results from the latter report excluding the influence of surgeons and having identified patient age as an independent risk factor.

Previous reports did not find any significant effect of gender and age on subsequent revision/removal rates [2, 4]. Nevertheless, in the presented study, the higher patient's age at primary operation was found to be statistically significant risk factor of subsequent implant revision, while gender had no significant impact. One study found that the only statistically significant indicator for an increased risk of revision was a smaller stem size [2]. These findings correlate well with our results, as most of the ABG-II femoral stems with subsequent periprosthetic fracture had preoperative proximal femoral stovepipe shape (Dorr type C) and postoperative non-tight femoral fill ratio < 0.80. Proximal femoral shape may therefore play an important role in the multifactorial risk setting for postoperative periprosthetic fractures.

The limitation of this report is retrospective evaluation of clinical outcome based entirely on subsequent revision procedures without additional clinical parameters, such as postoperative range of motion in the hip joint, residual pain, leg length discrepancy and functional performance. That does not interfere with our findings, as it describes the clinical data which are just a clinical consequence for patients and do not have an influence on the parameters of our study.

Our findings on risk factors for subsequent periprosthetic fracture concur with previous knowledge in this field. Medical achievements have led to patients surpassing previously established life expectancies; therefore, older and more active patients are nowadays undergoing arthroplasty and are therefore more prone to subsequent periprosthetic fracture [23]. Most injuries happen after low-energy trauma, e.g., falls from standing height, [24]. The average patient sustaining a periprosthetic femoral fracture is an elderly man or woman, with several concomitant chronic diseases. Osteoporosis is a well-known occurrence in the elderly, especially in women, that results in decreased bone mass, increased bone fragility and a higher propensity for fractures from low-energy trauma around total hip replacement implants. Bisphosphonates were found to be beneficial to decreasing periprosthetic bone loss after total hip arthroplasty and preventing periprosthetic fractures [25]. Because osteoporosis is most commonly found in post-menopausal women, as a result of decreasing levels of estrogen, estrogen hormone replacement therapy is an effective way of increasing bone mineral density and preventing osteoporotic fractures in post-menopausal women [26]. General muscle loss, weakness and loss of coordination also accompany old age, further increasing the risk of falls on even ground. Furthermore, the benzodiazepines that are often prescribed in the elderly for anxiety and insomnia are known to negatively impact coordination and predispose the patient to falls [27]. Older people are also at a higher risk of falling, because they are more likely to have balance problems, muscle weakness and vision loss related to age. Balance training, walking aids and removal of mats and slippery surfaces may be employed to improve overall strength and coordination, thereby reducing the chance of falls [24]. Last but not the least, the choice of implant fixation at the primary total hip arthroplasty has a significant impact on subsequent periprosthetic fracture risk. Intraoperative fractures maya occur up to 14 times more often with uncemented femoral stems [28], particularly with female patients over 65 years of age, and postoperative fracture risk is also increased by almost 3 times with uncemented femoral stem fixation [29]. If one intends to minimize these risks, it is therefore preferable to use cemented arthroplasty implants in the elderly with weakened bone.

## Conclusion

The study presents the largest published ABG-II femoral stem cohort from a single hospital with up to 10 years of follow-up with 9291 component-years of observation and surgeon-stratification. Considering the cumulative probability of periprosthetic fracture 2.1% at 1 year, 2.3% at 2 years, 3.2% at 5 years and 6.5% at 10 years after the implantation, this study found very high periprosthetic fracture risk of ABG-II that increased with patients’ age, had no variability between different surgeons and was considerably higher from other uncemented femoral stems used at the same hospital.

## Data Availability

Anonymized data of all included study subjects are available at the corresponding author upon request.
